# Computer-based monitoring of global cardiovascular dynamics during acute pulmonary embolism and septic shock in swine

**DOI:** 10.1186/cc10835

**Published:** 2012-03-20

**Authors:** JA Revie, DJ Stevenson, JG Chase, BC Lambermont, A Ghuysen, P Kolh, GM Shaw, T Desaive

**Affiliations:** 1 University of Canterbury, Christchurch, New Zealand; 2 University of Liege, Belgium; 3 Christchurch Hospital, Christchurch, New Zealand

## Introduction

Acute pulmonary embolism (APE) and septic shock (SS) are highly prevalent dysfunctions in the ICU due to the immunocompromised and immobile state of ICU patients. This research retrospectively tests the ability of a computer-based method to monitor acute hemodynamic changes in pigs. If proven, this method could assist ICU staff by providing a clear physiological, patient-specific picture of cardiovascular status for decision support.

## Methods

In two porcine studies, APE (*n *= 5) and SS (*n *= 4) were induced using autologous blood clots and endotoxin infusions. Hemodynamic measurements were recorded every 30 minutes for 4 hours (*n *= 80). Subject-specific cardiovascular models were identified from typical ICU measurements obtained from each of these datasets, including aortic and pulmonary artery pressure, stroke volume, heart rate, global end-diastolic volume, and mitral and tricuspid valve closure times. Model outputs and identified parameters were compared to experimentally derived indices, measurements not used in the identification process, and known trends to validate the accuracy of the models.

## Results

The models accurately predicted maximum ventricular pressures and volumes, not used in the identification process, to mean percentage errors of 7.1% and 6.7% (less than measurement error ~10%). Mean modelled pulmonary vascular resistances (PVR) compared well (*R*^2 ^= 0.81 for APE and *R*^2 ^= 0.95 for SS) to experimentally derived values. Importantly, in the APE study a 91% rise from baseline in the mean PVR was identified with an 89% increase seen in the SS pigs. Contrasting behaviour between the two studies was observed for systemic vascular resistance (SVR) with a maximum drop of 40% from baseline recorded at T120 for SS, indicating a loss of vascular tone as expected, where at the same time in the APE study the average SVR had increased by 13%. An increase in the ratio of right to left ventricle end volume was identified in all nine pigs, indicating right ventricular distension and a leftward shift in the intraventricular septum.

## Conclusion

These results indicate that subject-specific cardiovascular models are capable of tracking well-known global hemodynamic trends of two common forms of shock in the ICU. The method shows potential and could provide a means for continuous cardiovascular monitoring at little extra cost as no extra measurements or expensive devices are required.

